# Primary care team characteristics associated with video use: a retrospective national study at the Veterans Health Administration

**DOI:** 10.1186/s12875-024-02565-4

**Published:** 2024-09-07

**Authors:** Claudia Der-Martirosian, Caroline K. Yoo, W. Neil Steers, Cynthia G. Hou, Karen Chu, Jacqueline Ferguson, Maia Carter, Leonie Heyworth, Lucinda B. Leung

**Affiliations:** 1 Veterans Affairs Greater Los Angeles Healthcare System/Center for the Study of Healthcare Innovation, Implementation, & Policy (CSHIIP), Los Angeles, CA USA; 2 Veterans Emergency Management Evaluation Center (VEMEC), Department of Veterans Affairs, North Hills, CA USA; 3 Center for Innovation to Implementation, Veterans Affairs Palo Alto Health Care System, Menlo Park, CA USA; 4 Office of Primary Care/Patient Care Services, Veterans Health Administration, Washington, DC USA; 5 Office of Connected Care/Telehealth Services, Veterans Health Administration, Washington, DC USA; 6 Department of Medicine, University of California San Diego School of Medicine, San Diego, CA USA; 7 Department of Medicine, Division of General Internal Medicine & Health Services Research, UCLA David Geffen School of Medicine, Los Angeles, CA USA

**Keywords:** Telehealth, Video use, Primary care teams, Provider characteristics, Veterans health administration

## Abstract

**Objective:**

To examine primary care (PC) team members’ characteristics associated with video use at the Veterans Health Administration (VA).

**Methods:**

VA electronic data were used to identify PC team characteristics associated with any video-based PC visit, during the three-year study period (3/15/2019-3/15/2022). Multilevel mixed-effects logistic regression models on repeated yearly observations were used, adjusting for patient- and healthcare system-level characteristics, and study year. We included five PC team categories: 1.PC providers (PCP), which includes physicians, nurse practitioners, physician assistants, 2.Nurses (RN/LVN/LPN/other nurses), 3.Mental health (MH) specialists, 4.Social workers (SW), and 5.Clinical pharmacists (PharmD).

**Population:**

54,494 PC care team members nationwide (61,728,154 PC visits; 4,916,960 patients), including 14,422 PCPs, 30,273 nurses, 2,721 MH specialists, 4,065 SWs, and 3,013 PharmDs.

**Results:**

The mean age was 46.1(SD = 11.3) years; 77.1% were women. Percent of video use among PC team members varied from 24 to 84%. In fully adjusted models, older clinicians were more likely to use video compared to the youngest age group (18–29 years old) (example: 50–59 age group: OR = 1.12,95%CI:1.07–1.18). Women were more likely to use video (OR = 1.18, 95%CI:1.14–1.22) compared to men. MH specialists (OR = 7.87,95%CI:7.32–8.46), PharmDs (OR = 1.16,95%CI:1.09–1.25), and SWs (OR = 1.51,95%CI:1.41–1.61) were more likely, whereas nurses (OR = 0.65,95%CI:0.62–0.67) were less likely to use video compared to PCPs.

**Conclusions:**

This study highlights more video use among MH specialists, SWs, and PharmDs, and less video use among nurses compared to PCPs. Older and women clinicians, regardless of their role, used more video. This study helps to inform the care coordination of video-based delivery among interdisciplinary PC team members.

**Supplementary Information:**

The online version contains supplementary material available at 10.1186/s12875-024-02565-4.

## Introduction

The rapid expansion of video-based telehealth services in primary care (PC) since the onset of the COVID-19 pandemic [1–7] has provided new opportunities to examine a wide range of virtual care topics. Topics include patient and clinician satisfaction with services [8–11], advantages and disadvantages to implementation and use of telehealth services [12–19], patient and clinicians’ characteristics of telehealth use [20–26], disparities in access to video visits [27–30], and the extent to which telehealth can be incorporated in routine primary care [31–35]. Most of these small size studies have shown acceptability and satisfaction of video use among clinicians [8–10, 12, 13, 36], however, we are not certain the extent to which clinicians in PC use video-based services on large scale.

One important aspect of virtual care coordination among interdisciplinary, team-based PC settings is to have a better understanding about PC team members’ characteristics associated with the use of telehealth services. However, there is a knowledge gap about the extent to which different PC team members, such as physicians, nurse practitioners, physician assistants, other nurses, mental health providers, clinical pharmacists, and social workers, exercise their preferences and actually utilize video-based care, as well as its association with other PC team characteristics (e.g., gender, age).

To address this gap, we conducted a national study of the provision of video-based care among interdisciplinary team-based PC team members. The Veterans Health Administration (VA) is an ideal place to conduct this study, since it has patient-aligned care teams with interdisciplinary team members in PC, which are similar to patient-centered medical models at various non-VA settings. Additionally, like many healthcare settings, access to telehealth services in PC increased dramatically at the VA immediately after onset of COVID-19 [5, 37–41]. Moreover, VA was an early adopter and a national leader [42] in telehealth, with over two decades of experience in video-based care [5].

In this study, we defined telehealth as using technology for a remote medical synchronous video encounter [43] and examined PC team members’ characteristics associated with use of video for primary care visits among different types of clinicians who care for the same patient population, after controlling for patient- and healthcare system-level characteristics. We also identified PC team members’ demographic characteristics, such as age and gender, that are associated with video use in PC.

## Methods

### Study design and study sample

This was a retrospective cohort study of VA interdisciplinary PC team members (e.g., physicians, nurses, social workers) from 12-months before and 24-months after the COVID-19 onset (March 16, 2019, through March 15, 2022) at 138 VA healthcare systems (i.e., VA medical center and associated community clinics) nationwide. We excluded two healthcare systems (one healthcare system that had transitioned to the Cerner Millennium EHR system and did not have data for the entire study period, and another healthcare system in a foreign country). VA patients who had at least one PC visit during the year prior to COVID-19 onset (March 16, 2019, through March 15, 2020) were included in the study. Characteristics of their PC visits (e.g., telehealth modality) during the 24-month period post COVID-19 onset (March 16, 2020, through March 15, 2022) and associated PC visit team members were identified. The study sample included 54,494 PC team members, 4,916,960 patients, and 61,728,154 PC visits. The study was part of an ongoing VA quality improvement effort approved by the VA Greater Los Angeles Healthcare System institutional review board, which deemed the study as non-human participants research and waived the informed consent requirements.

### Study data

VA electronic records were used to compile patient-, PC team-, and healthcare system-level administrative, clinical, and utilization data. All data management and analyses were conducted within the VA Informatics and Computing Infrastructure. Patient-level demographic and clinical characteristics, outpatient visit modality and its dates, and PC team member types were from the VA Corporate Data Warehouse. Rurality of patient residence was determined from the Geospatial Service Support Center geocoded enrollee files. PC team members’ demographic characteristics were obtained from the Personnel and Accounting Integrated Data payroll system. Lastly, healthcare system-level characteristics were obtained from the VHA Support Service Center.

### Study measures

In this study, we examined video-based PC visits, which are a telehealth modality that allows synchronous communication between patients and PC team members, using a camera-enabled device. For each completed PC visit, clinic codes and Current Procedural Terminology (CPT) modifier for synchronous telemedicine service were used to identify video visits (see Appendix A). For each patient, the most frequently seen PC team member for PC visits during the study year was assigned as the main clinician. Lastly, PC team members were assigned to a healthcare system in which they were most frequently associated during each study year. In the case of ties, PC team members were assigned to the healthcare system in which they provided their most recent encounter. It should be noted that even though there is a large amount of telephone use in VA primary care, the data accessible for this study could not decipher between short follow-up calls (e.g., lab test results), that are routinely conducted by primary care team members, and ‘real’ synchronous telephone visits (e.g., lets discuss your medication plan), that are comparable to synchronous video visits. As such, we focused on video-based care.

PC team characteristics included five age categories (18–29, 30–39, 40–49, 50–59, 60+), gender (women, men), and five PC team type categories. PC team types were based on position titles and included: (1) Primary care providers (PCPs), which included physicians, nurse practitioners, and physician assistants, (2) nurses, which included licensed vocational nurses, licensed practical nurses, registered nurses, and other nurses, (3) mental health specialists (psychologists, psychiatrists, and mental health counselors), (4) clinical pharmacists, and (5) social workers.

Patient demographic characteristics known to be associated with video use [26] were included as covariates: patient’s age (18–44, 45–64, 65–75, 75+), gender (women or men), race/ethnicity (non-Hispanic White, non-Hispanic Black, Hispanic, non-Hispanic other minority, unknown), marital status (married, divorced/widowed, single/never married), and the Charlson Comorbidity Index categories (0, 1, 2+) [44]. The rurality of patient’s residence (rural/highly rural, urban) is based on the Rural Urban Commuting Area (RUCA) classifications [45]. VA enrollment priority groups, which assigns patients to one of the eight priority groups, were also included in the analysis. This measure is based on military service-related disability, income, and other criteria and was further grouped into four categories: high disability, low/moderate disability, low income, and enrolled without special considerations [46]. High disability group refers to having > 50% service-related disability or catastrophically disabled. [47] Patients are deemed catastrophically disabled based on a VA clinical decision when they suffer from a severely disabling injury, disorder, or disease that permanently impairs their ability to perform activities of daily living to such an extent that they require personal or mechanical assistance to leave their home or bed, or constant supervision to prevent physical harm to themselves or others. [47] Low/moderate disability group includes 10–40% service-related disability or military exposures. Low income includes Veterans having household income below geographically adjusted threshold [48]. The enrolled without special considerations group refers to 0% service-related disability and co-pay requirement [47, 48].

Healthcare system characteristics included facility complexity and rurality [49]. Facility complexity was categorized as: low, medium, and high. This measure was defined by the VA Facility Complexity Model based on multiple criteria, which includes volume, patient risk, teaching and research status, breadth of physician specialties, and intensive care unit (ICU) level [50]. Rurality of the healthcare system (rural vs. urban) was designated based on rurality of the majority of the associated hospital and clinics, following the RUCA classifications [45]. Medical centers and clinics were categorized as rural if their RUCA classifications were rural, highly rural, or insular.

### Statistical analyses

For bivariate analysis, unadjusted percentages of any video use were calculated by PC team characteristics (age categories, gender, and provider type) for each study year (one year before COVID-19 onset, one year after COVID-19 onset, and two years after COVID-19 onset). For multivariate analysis, PC team-level predictors of any video use were examined after controlling for study year, patient- and healthcare system-level characteristics, using multilevel mixed-effects logistic regression models. Random intercepts for patients and PC team were used to account for patient/PC team clustering.

All statistical tests were two-sided at the significance threshold of *p* < 0.05. Data were analyzed in Stata version 17.0 (StataCorp, College Station, TX) from August 2022 to February 2023.

## Results

The study included 14,422 (26.5%) PCPs, 30,273 (55.6%) nurses, 2,721(5.0%) mental health specialists, 3,013 (5.5%) clinical pharmacists, and 4,065 (7.5%) social workers. Interdisciplinary PC team members had a mean age of 46.1 years [SD 11.3], and 77.1% were women (Table 1).

**Table 1:** Baseline characteristics of VA Primary Care (PC) Team Members, Nationwide*

PC team members’ characteristics	*N* (%) of PC team members *N* = 54,494
Age, Mean (SD)	46.1 (SD = 11.3)
Age Categories, n (%)	
18–29	3,878 (7.1%)
30–39	13,473 (24.7%)
40–49	15,310 (28.1%)
50–59	14,451 (26.5%)
60+	7,382 (13.5%)
Female, n (%)	42,030 (77.1%)
Provider Type: n (%)	
Primary Care Providers (MD, NP, PA)	14,422 (26.5%)
Nurses (LVN, LPN, RN)	30,273 (55.6%)
Mental Health Specialists	2,721 (5.0%)
Clinical Pharmacists	3,013 (5.5%)
Social workers	4,065 (7.5%)

*138 VA Healthcare Systems; Baseline: One-year before COVID-19 onset (3/16/2019-3/15/2020)

In unadjusted analyses, the percentages of VA PC team members with any video visit ranged from 24.0 to 48.8% for one year before COVID-19, followed by 69.9–82.5% during the first COVID-19-year, and then 65.9–83.8% during the second COVID-19-year. PC team members in the youngest age category used video the least compared to PC team members in the oldest age category (24.0% for 18–29 years old vs. 34.6% for 60 + years old) one year before COVID-19. The same pattern persisted during the first and second COVID-19-years (see Table 2). A higher proportion of men had at least one video-based visit compared to women PC team members (37.6% men vs. 32.8% women) before COVID-19. However, during the first COVID-19-year, women had slightly more video-based visits (74.4% men vs. 75.9% women), followed by a reversal, where a higher proportion of men had video-based visits (73.7% men vs. 68.1% women) in the second COVID-19-year. Before COVID-19, PCPs (48.8%) and mental health specialists (42.1%) had the highest percent with any video-based visit, followed by clinical pharmacists (38.7%), social workers (26.5%) and nurses (24.8%). In the first COVID-19-year, video use increased sharply for all PC team types. However, in the second COVID-19-year, video use increased for PCPs, mental health specialists, and social workers, but it decreased for nurses and clinical pharmacists (see Table 2).

**Table 2:** Unadjusted percentage of any video use among VA PC Team members by PC Team characteristics

PC Team Characteristics	Percent of PC team members with any video-based visit			
	One-year before COVID-19 onset *N* = 34,573	One-year after COVID-19 onset *N* = 38,248	Two-years after COVID-19 onset *N* = 38,803	Overall *N* = 54,494
**Age categories**				
18–29	24.0%	69.9%	65.9%	62.2%
30–39	32.5%	73.6%	68.1%	65.5%
40–49	34.3%	74.9%	68.8%	67.6%
50–59	36.0%	76.4%	70.8%	69.3%
60+	34.6%	75.2%	72.3%	63.7%
**Gender**				
Female	32.8%	74.4%	68.1%	66.7%
Male	37.6%	75.9%	73.7%	66.5%
**Provider type**				
Primary Care Providers	48.8%	82.5%	83.8%	73.1%
Nurses	24.8%	70.3%	60.2%	63.7%
Mental Health Specialists	42.1%	82.3%	85.7%	68.2%
Clinical Pharmacists	38.7%	78.6%	77.3%	70.6%
Social Workers	26.5%	70.7%	70.9%	61.3%

*Notes* One-year before COVID-19 onset (3/16/2019-3/15/2020), one-year after COVID-19 onset (3/16/2020-3/15/2021), two-years after COVID-19 onset (3/16/2021-3/15/2022), overall (3/16/2019-3/15/2022). The proportion of in-person visits can be calculated by subtracting each value (% any video-based visit) from 100%. For example, for age category 18–29 (one-year before COVID-19), 76% provided in-person primary care

**Fig. 1:**
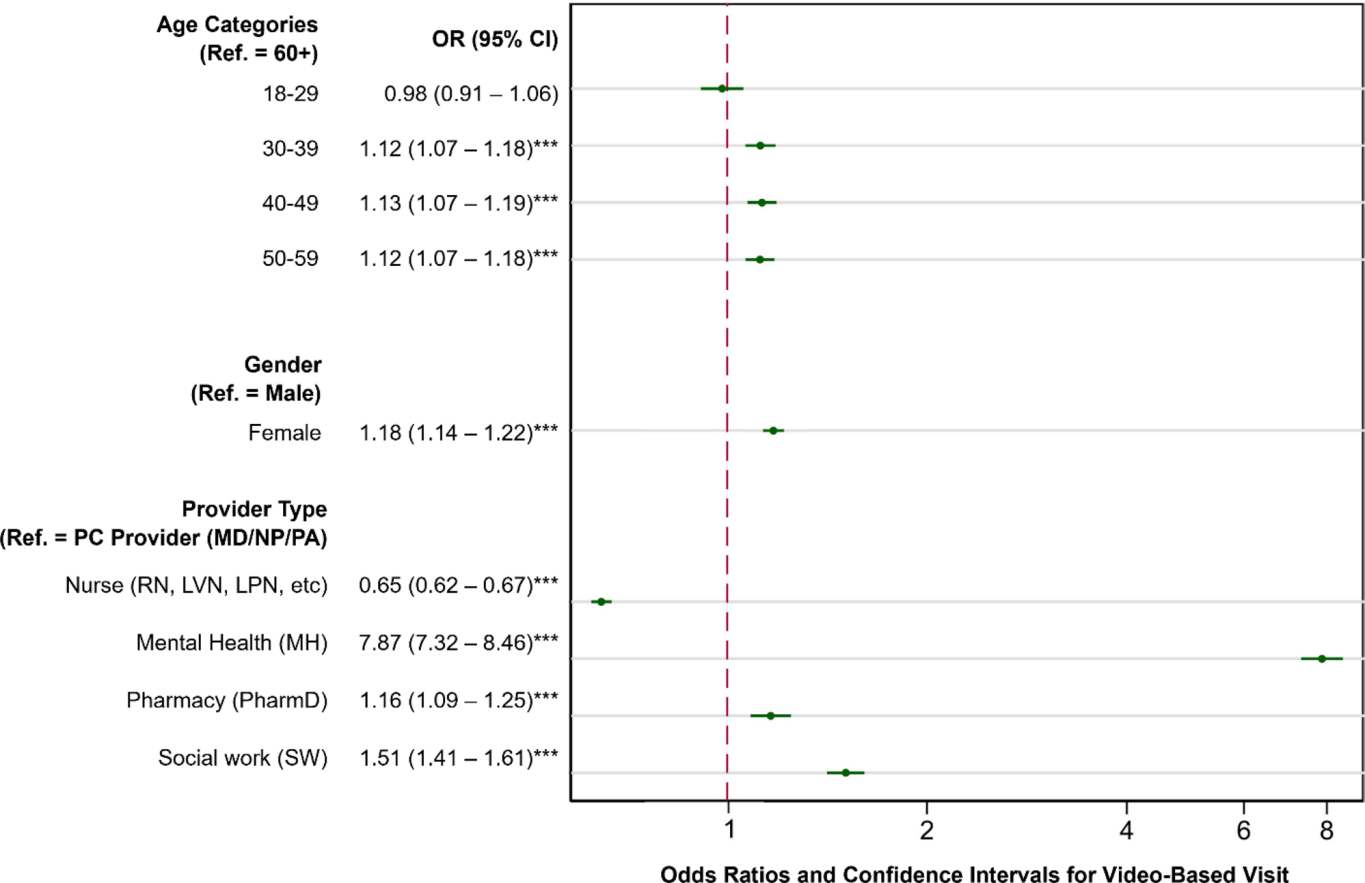
Adjusted odds ratios for primary care (PC) video-based visits by PC team characteristics using multilevel mixed effects, logistic regression. *Note* The regression model adjusted for patient- and healthcare system-level characteristics (patient: socio-demographic and clinical characteristics; healthcare system: facility complexity and rurality), as well as indicators for regional networks of care and study year. *Abbreviations* Odds ratio (OR); 95% confidence interval (95% CI). *p < 0.05, **p < 0.01, ***p < 0.001

After adjusting for patient- and healthcare system-level characteristics and study year, multilevel logistic regression models of video-based care indicated that older PC team members were more likely to have any video-based visit compared to the youngest age group category (18–29 years old) (OR = 1.12, 95% CI: 1.07–1.18 for 30–39 years old; OR = 1.13, 95% CI: 1.07–1.19 for 40–49 years old; OR = 1.12, 95% CI: 1.07–1.18 for 50–59 years old; p’s < 0.001) (see Fig. 1).

In fully adjusted models, however, women were more likely to use video (OR = 1.18, 95% CI:1.14–1.22; *p* < 0.001) compared to men. Regarding PC team types, mental health specialists (OR = 7.87, 95% CI: 7.32–8.46; *p* < 0.001), social workers (OR = 1.51, 95% CI: 1.41–1.61; *p* < 0.001), and clinical pharmacists (OR = 1.16, 95% CI: 1.09–1.25; *p* < 0.001) were found to use more video compared to PCPs. Nurses were found to use less video (OR = 0.65, 95% CI: 0.62–0.67; *p* < 0.001) compared to PCPs. For complete regression results with odds ratios and 95% CI for patient-, PC team- and healthcare system-level characteristics see Table 3.

**Table 3:** Odds ratios for having a video-based visit in primary care by patient-, PC team-, and healthcare system-level characteristics, using multilevel mixed-effects logistic regression

	OR (95% CI)
**Patient-level**	
Year (ref: 1-year before COVID-19 onset)	
1st year after COVID-19 onset	40.4 (39.8–41.0) ***
2nd year after COVID-19 onset	27.8 (27.4–28.2) ***
Age categories (ref: 75+)	
18–44	3.40 (3.36–3.44) ***
45–64	2.63 (2.61–2.66) ***
65–75	1.50 (1.49–1.51) ***
Female (ref: Male)	1.47 (1.45–1.48) ***
Race/ethnicity (ref: Non-Hispanic White)	
Non-Hispanic Black	1.03 (1.02–1.04) ***
Hispanic	1.00 (0.98–1.01)
Non-Hispanic Other	0.97 (0.95–0.98) ***
Unknown	0.92 (0.90–0.93) ***
Marital status (Ref: Single/Never married)	
Married	1.19 (1.19–1.20) ***
Divorced/Widowed	1.08 (1.07–1.09) ***
Rurality of patient residence	0.95 (0.94–0.96) ***
Charlson Comorbidity Index (CCI) (ref: 0)	
1	1.09 (1.09–1.10) ***
2+	1.19 (1.18–1.20) ***
Enrollment priority group (Ref: Enrolled without special considerations)^a, b^	
High disability	1.32 (1.31–1.33) ***
Low/moderate disability	1.14 (1.13–1.15) ***
Low income	0.91 (0.90–0.92) ***
**PC Team-level**	
Age categories (ref: 60+)	
18–29	0.98 (0.91–1.06)
30–39	1.12 (1.07–1.18) ***
40–49	1.13 (1.07–1.19) ***
50–59	1.12 (1.07–1.18) ***
Female (ref: Male)	1.18 (1.14–1.22) ***
Provider Type (ref: PC Providers)	
Nurses	0.65 (0.62–0.67) ***
Mental Health Specialists	7.87 (7.32–8.46) ***
Clinical Pharmacists	1.16 (1.09–1.25) ***
Social workers	1.51 (1.41–1.61) ***
**Healthcare System-level** ^**c**^	
Service complexity level (ref: Low)^d^	
High	1.32 (1.25–1.40) ***
Medium	1.33 (1.26–1.42) ***
Rurality of healthcare system (ref: urban)	0.83 (0.78–0.89) ***

^a^ High disability refers to having > 50% service-related disability or catastrophically disabled. The determination of catastrophic disability is based on a combination of diagnostic codes and clinical evaluation and judgment. This approach allows for flexibility and a comprehensive assessment of individual cases, as diagnostic codes alone may not fully capture the extent of a disability. Low/moderate disability includes 10–40% service-related disability or military exposures

^b^ Low income includes patients having an annual income below area-adjusted income threshold. Enrolled without special considerations refers to 0% service-related disability and co-pay requirement. More specifically, income data for patients are systematically collected during VA health benefits enrollment process. As part of this process, Veterans submit an application where they provide detailed income and net worth information, which serves as a basis for eligibility determination. To ensure continued eligibility and account for changes in financial status, patients must also update their income information on an annual basis. The VA sets income limits by utilizing the Means Test Thresholds and the Geographically Adjusted Income Limits (GMT), which are based on the household size and the residential ZIP code of each Veteran. These thresholds are adjusted annually and are aligned with the U.S. Department of Housing and Urban Development’s low-income limits for the same geographic areas. The alignment with these federal low-income limits ensures that the thresholds are based on nationally standardized measure across different regions

^c^ Indicators for regional networks of care, known as Veterans Integrated Service Network (VISN), were included in the regression model as fixed effects but are not shown in the table

^d^ High complexity facilities have high levels of patient volume, patient risk, and teaching/research activities. Medium complexity refers to medium levels of patient volume, medium-risk patients, and less teaching/research activities. Low complexity refers to small patient volume, low patient risk, and little to no teaching/research activities

*Abbreviations* Odds ratio (OR); 95% confidence interval (95% CI)

*p < 0.05, **p < 0.01, ***p < 0.001

## Discussion

To our knowledge, this is the first national multi-year study to examine the association between healthcare providers’ characteristics and real-time synchronous video visits among interdisciplinary PC teams. Previous research on provider characteristics associated with the use of video-based care mainly focused on providers’ preferences of using video-based care [9, 14, 16–18, 22], their satisfaction with video-based care [8–11], and the benefits and challenges of using video-based care [12, 13, 15–17, 19], often using small sample sizes. Consistent with prior research [23], PC team member type, such as PC providers, nurses, and mental health specialists, was an important predictor of video use. Similar to previous findings, after controlling for relevant patient- and healthcare system-level characteristics, mental health specialists, social workers, and clinical pharmacists in PC were more likely, while nurses were less likely, to use video compared to PC providers (such as physicians, nurse practitioners, and physician assistants). Identification of what types of PC team members are more likely to provide video-based care illustrates “exercised preference” – i.e., who is actually willing to conduct video visits. These findings can help guide the care coordination of video visits among PC team members. Given that mental health specialists, social workers, and clinical pharmacists do not conduct physical examinations during clinical visits, it is not surprising that they were more likely to use video-based care compared to PCPs. Conversely, it is not surprising that nurses were less likely to use video compared to PCPs, given that nurses in PC settings are often tasked with checking patient’s vital signs (during in-person/clinic visits), triaging patients (via phone calls), or sharing lab results (via phone calls). These findings, like those in previous studies [51, 52], further illustrate that the appropriateness of a video-based PC visit depends on the types of services that are being provided.

Regarding provider demographic characteristics, the study illustrated that older providers were more likely to use video compared to the youngest providers (18–29 years old). Age might be a proxy for clinical experience. Even though younger PC members are perhaps more tech-savvy, older PC team members might have more clinical experience and established patient panels and perhaps have more flexibility in teleworking options or administrative positions, where video visits become more feasible. The study findings also illustrated that women were more likely to use video compared to men. This finding may be because women having more family obligations at home, where teleworking may be a viable option for these clinicians. In addition to provider characteristics, providers try to meet their patients where they are at by being flexible with the type of visit (in-person or virtual) they offer or schedule. Patient’s preferences, and/or other factors (such as lack of transportation), may influence whether the patient is able to attend in-person or video visits.

The major strengths of this study are the use of national data and identification of PC team characteristics of video-use among interdisciplinary primary care team members, after controlling for the effects of patient- and healthcare system-level characteristics. However, this study has several limitations. First, the scope of PC team characteristics is limited to three variables: PC team type, age, and gender, which were accessed using electronic administrative data. The gender variable included men or women and did not include other gender identities (e.g., non-binary, transgender). Future studies might have opportunities to include additional data on patients and providers who identify with other gender identities. There are many other PC team characteristics that could impact video use, such as length of employment at the VA, clinical experience, and provider preference or comfort. Unfortunately, additional PC team variables were not available in these records. Therefore, more research should be conducted to assess the association of additional clinician characteristics with video use. Second, since the data available for this study could not decipher between short follow-up telephone visits vs. ‘real’ synchronous telephone visits, this study only focused on video-based care. Furthermore, provider characteristics related to telephone care are likely distinct from those associated with video-based care given that telephone-based care has been commonplace for a longer time and relies on near ubiquitous phone technology with minimal requirements. Whereas video-based care has experienced a surge in adoption recently following the COVID19 pandemic and still faces challenges for more widespread adoption. Third, generalizability of the study findings from VA to non-VA healthcare settings might be limited. However, recent COVID-19 telehealth waivers have increased non-VA healthcare providers’ video-based care capability. Therefore, today there are more similarities than ever between VA and non-VA telehealth services. As such, study findings may still be applicable to non-VA clinical settings and contribute to the growing evidence base surrounding provider characteristics associated with video use.

## Conclusion

In addition to patient- and healthcare system-level characteristics, it is also important to consider PC team members’ characteristics that are associated with video telehealth use. This study highlights the significant association of PC team type, age, and gender with video use, after taking into account patient- and healthcare system-level characteristics. This fills in the knowledge gap on clinician characteristics of video use, using national data. By focusing on PC team characteristics, this study helps to inform the care coordination of video-based visits among interdisciplinary primary care team members.

## Electronic supplementary material

Below is the link to the electronic supplementary material.

Supplementary Material 1

## Data Availability

To protect study participant privacy, the study data cannot be shared openly.
