# Supplementary material for: Primary care team characteristics associated with video use: a retrospective national study at the Veterans Health Administration
Source: BMC Prim Care. 2024 Sep 7;25:333. doi: 10.1186/s12875-024-02565-4 (PMC11380331; doi:10.1186/s12875-024-02565-4)
Supplement: Supplementary file 1 — Supplementary Material 1 [file 12875_2024_2565_MOESM1_ESM.docx]

**Appendix A.** Clinic Codes and Current Procedural Terminology (CPT) Modifier for Primary Care and Video-Based Visits

| **Clinic Code or Current Procedural Terminology (CPT) modifier** | **Definition** | **Primary Care** | **Video** |
| --- | --- | --- | --- |
| 322 | Comprehensive Women’s Primary Care Clinic | X |  |
| 323 | Primary Care Medicine | X |  |
| 338 | Telephone Primary Care | X |  |
| 348 | Primary Care Shared Appointment | X |  |
| 350 | Geriatric Patient Aligned Care Team | X |  |
| 531 | Primary Care for Patients with Serious Mental Illness (SMI) | X |  |
| 534 | Mental Health Integrated Care - Individual | X |  |
| 539 | Mental Health Integrated Care - Group | X |  |
| 704 | Women’s Gender-Specific Preventive Care | X |  |
| 179 | Real Time Clinical Video Telehealth to Home- Provider Site |  | X |
| 648 | Real Time Clinical Video Telehealth with Non-VA Medical Center Location- Provider Site |  | X |
| 679 | National Center Real Time Clinical Video Telehealth to Home- Provider Site |  | X |
| 95 | Synchronous telemedicine service rendered via a real-time interactive audio and video telecommunications system |  | X |
